# Quantitative Determination of *p*-Cymene, Thymol, Neryl Acetate, and *β*-Caryophyllene in Different Growth Periods and Parts of *Eupatorium fortunei* Turcz. by GC-MS/MS

**DOI:** 10.1155/2021/2174667

**Published:** 2021-08-02

**Authors:** Guanjun Nan, Lina Zhang, Zhengzheng Liu, Yu Liu, Yan Du, Hongwen Zhao, Hongxia Zheng, Rong Lin, Guangde Yang, Shaohua Zheng

**Affiliations:** ^1^School of Pharmacy, Xi'an Jiaotong University, Xi'an 710061, China; ^2^Department of Pharmacology, Xi'an Jiaotong University, Xi'an 710061, China; ^3^Department of Anesthesiology and Operation, The First Affiliated Hospital of Xi'an Jiaotong University, Xi'an 710061, China

## Abstract

*Eupatorium fortunei* Turcz. is a widely used Chinese herbal medicine in China. In this study, a gas chromatography-triple quadrupole mass spectrometry (GC-MS/MS) method was developed and validated to simultaneously determine the contents of *p*-cymene, thymol, neryl acetate, and *β*-caryophyllene in roots, stems, and leaves of *Eupatorium fortunei* Turcz. harvested at different growth periods. All four constituents could be detected in leaves, three could be detected in stems except *β*-caryophyllene, and only thymol could be detected in roots. The order of the total contents of four constituents in different parts was leaves > stems > roots. It indicated that the leaves could be the proper medicinal parts of *Eupatorium fortunei* Turcz. The content of four constituents in leaves varied a lot among different growth periods and showed an M-shaped change trend with the growth of *Eupatorium fortunei* Turcz. The four constituents accumulated to the highest values in early July followed by mid-September. Accordingly, the best harvest time of *Eupatorium fortunei* Turcz. is early July and mid-September.

## 1. Introduction

*Eupatorium fortunei* Turcz., one species of the genus *Eupatorium* (Compositae), is a commonly used aromatic traditional herbal medicine in China. It has been applied for relieving nausea, vomiting, and poor appetite due to dampness obstruction since thousands of years ago [1]. Previous studies showed that the plant contains a number of bioactive natural products [2, 3]. The content determination of total volatile oil in *Eupatorium fortunei* Turcz. is stipulated in Chinese Pharmacopeia, and the total volatile oil is consequently used as an important index to evaluate its quality [4], while various studies showed that constituents in the volatile oil of *Eupatorium fortunei* Turcz. from different regions and harvest time varied a lot [5–7]. Accordingly, it is of great significance to assay representative constituents in the volatile oil of *Eupatorium fortunei* Turcz.

**Figure 1:**
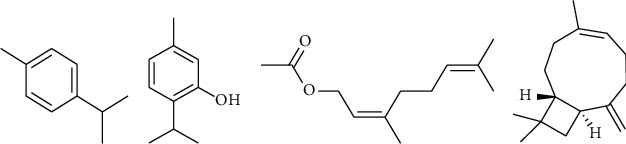
Chemical structures of (a) *p*-cymene, (b) thymol, (c) neryl acetate, and (d) *β*-caryophyllene.

Based on the related literature, *p*-cymene and neryl acetate were the earliest separated and identified compounds from the volatile oil of *Eupatorium fortunei* Turcz. [8]. It has been reported that *p*-cymene possesses an effect on dispelling phlegm [9]. Together with neryl acetate, it was also claimed to be the main active ingredient of the volatile oil for the treatment of influenza [10]. *β*-Caryophyllene was reported to be the constituent with the highest content in the volatile oil and has been studied as an indicator constituent to establish the content determination method of *Eupatorium fortunei* Turcz. [11, 12]. Thymol and its derivatives are abundant in the plant [2, 13–15]. It could be concluded that the contents of *p*-cymene, thymol, neryl acetate, and *β*-caryophyllene (structured as shown in Figure 1) have a significant effect on the quality of *Eupatorium fortunei* Turcz.

It is well known that traditional Chinese medicines have the characteristic of complex composition. The composition and content of compounds are closely related to the quality of traditional Chinese medicine. The growth period is always an important factor affecting the composition and content of constituents in the medicinal herbs. Thus, the appropriate harvesting time for traditional Chinese medicine is crucial to the formation of high-quality traditional Chinese medicine [16]. A lot of works have been performed on the effect of the developmental stage on the content of biologically active compounds in plants [17–19]. Accordingly, the quantities of the volatile oil in *Eupatorium fortunei* Turcz. may change during the growth stages. It is essential to study the content change of the volatile oil with growth for ensuring the quality of *Eupatorium fortunei* Turcz. to prevent and treat disease.

Therefore, in this work, *Eupatorium fortunei* Turcz. was harvested at different growth periods, and the contents of *p*-cymene, thymol, neryl acetate, and *β*-caryophyllene in different parts were determined by using GC-MS/MS. The dynamic characteristic and distribution of four constituents were analyzed.

## 2. Materials and Methods

### 2.1. Plant Materials

*Eupatorium fortunei* Turcz. was collected every fifteen days from April 15 to November 1, 2019, at the medicinal botanical garden of Xi'an (34°25' N, 108°98' E) and identified by Prof. Xiaofeng Niu. Ten plants were harvested at each stage. Each plant was separated into leaves, stems, and roots. Then, the leaves, stems, and roots were separately collected together and dried.

### 2.2. Reagents and Chemicals

Ethanol, ethyl acetate, petroleum ether (60°C∼90°C), and hexane were of analytical grade. *p*-Cymene (purity: 99.5%) and neryl acetate (purity: 95%) were purchased from Shanghai Aladdin BioChem Technology Co., Ltd. (Shanghai, China). Thymol (purity: 99%) and *β*-caryophyllene (purity: 90%) were purchased from J&K Scientific Ltd. (Beijing, China).

### 2.3. GC-MS/MS Analysis

A Shimadzu GC-MS 8040 equipped with SH-Rxi-5Sil MS capillary column (30 m, 0.25 mm i.d., 0.25 *μ*m film thickness) and triple quadrupole mass spectrometer was used (Japan). The helium of high purity (99.9999%) was the carrier gas at a flow rate of 1 mL·min^−1^. Samples were injected in a split mode with a ratio of 10 : 1. The temperature procedure of the column was set at 70°C for 3 min, increased to 80°C at a rate of 10°C·min^−1^, to 100°C at a rate of 20°C·min^−1^, to 130°C at a rate of 2°C·min^−1^, and to 280°C at a rate of 20°C·min^−1^, and maintained for 2 min. An electron ionization system was used with ionization energy of 70 eV. Injection, interface, and ionization temperature were set at 250°C, 250°C, and 230°C, respectively. We used standard compounds of each volatile under the MRM mode to assay the contents of four target components [20].

### 2.4. Preparation of Standard Solution and Samples

Each standard stock solution was prepared separately by dissolving accurate amount of compound in hexane. A series of working solutions of these 4 compounds were freshly prepared by diluting the mixed standard solution with hexane.

Dried leaves, stems, and roots of *Eupatorium fortunei* Turcz. were milled to powder by using a high-speed disintegrator, and 0.2 g ground powder was extracted with 20 mL hexane in an ultrasonic waterbath for 60 min. Additional hexane was added to make up the lost weight [16]. Then, the solution was filtered through a 0.22 *μ*m filter before GC-MS/MS analysis.

### 2.5. Statistical Analysis

All measurements were performed using triplicate samples. Data were expressed as mean ± SD. All data were statistically processed using GraphPad Prism 7 (GraphPad Software, CA, USA) and analyzed by Tukey's multiple comparisons test to calculate the difference significance at a 5% level (*p* < 0.05).

## 3. Results and Discussion

### 3.1. Optimization of GC-MS Analysis Conditions and Method Validation

**Figure 2:**
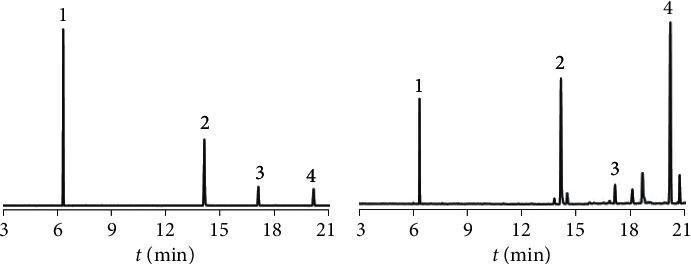
The MRM chromatogram of (a) references and (b) samples (1, *p*-cymene; 2, thymol; 3, neryl acetate; 4, *β*-caryophyllene).

Temperature procedure, parameters of ion pairs, and collision voltage are the key parameters for the GC-MS/MS method. These parameters were optimized in the present study. MRM was used for quantitative purposes with one target ion pair and two reference ion pairs. The corresponding collision voltage was optimized and is listed in Table 1. The MRM chromatogram is shown in Figure 2.

Linearity, range, accuracy, precision, limits of detection (LOD), and limits of quantification (LOQ) were evaluated under the optimal conditions (Table 2). *p*-Cymene, thymol, neryl acetate, and *β*-caryophyllene could be determined in a linear range of 0.01 *μ*g·mL^−1^∼0.38 *μ*g·mL^−1^, 0.05 *μ*g·mL^−1^∼6.21 *μ*g·mL^−1^, 0.11 *μ*g·mL^−1^∼0.88 *μ*g·mL^−1^, and 0.13 *μ*g·mL^−1^∼2.19 *μ*g·mL^−1^ with good correlation coefficients (*r* > 0.999), respectively. LODs and LOQs were calculated at a signal-to-noise of 3 and 10. Accuracy was measured by recoveries. The percentage recoveries were 105.31%, 106.41%, 99.61%, and 102.24% for *p*-cymene, thymol, neryl acetate, and *β*-caryophyllene, respectively. The repeatability of the method was determined by analyzing the standard solution 6 times. The relative standard deviations (RSD) for *p*-cymene, thymol, neryl acetate, and *β*-caryophyllene were 1.00%, 2.72%, 1.55%, and 1.59%, respectively. Six homogeneous samples were prepared and analyzed to evaluate the precisions. The RSD values of four analytes varied from 0.98% to 3.38%. For stability, the same sample was assayed separately at 0, 2, 4, 6, 8, and 12 h after extraction. The RSD values were less than 1.36%.

The results demonstrated that the developed method was accurate and repeatable which could be a reliable tool for identification and quantification of *p*-cymene, thymol, neryl acetate, and *β*-caryophyllene in *Eupatorium fortunei* Turcz.

### 3.2. Optimization of Sample Process Conditions

The influence factors, including extraction solvent, method, and duration were investigated through single-factor experiments. Extraction solvents including ethanol, ethyl acetate, petroleum ether (60°C∼90°C), and hexane were compared, and hexane resulted in a preferable peak and baseline. Two classical extraction methods, ultrasonic extraction, and reflux, were investigated. The ultrasonic extraction showed better extraction efficiency. Then, we set the extraction duration at 15 min, 30 min, 45 min, and 60 min. The contents of compounds were increased with the prolongation of extraction time (Table 3). Therefore, the extraction was conducted by using hexane in an ultrasonic waterbath for 60 min.

### 3.3. Distribution of Four Constituents in Different Parts of Eupatorium fortunei Turcz

Samples from different growth periods and parts were assayed. The contents of *p*-cymene, thymol, neryl acetate, and *β*-caryophyllene in different parts of *Eupatorium fortunei* Turcz. are listed in Table 4.

The contents of *p*-cymene, thymol, neryl acetate, and *β*-caryophyllene in leaves were ranged from 1.51 ± 0.056 to 25.25 ± 2.600 *μ*g·g^−1^, 20.81 ± 0.688 to 132.21 ± 3.169 *μ*g·g^−1^, 13.77 ± 2.600 to 59.90 ± 1.006 *μ*g·g^−1^, and 13.36 ± 2.611 to 144.21 ± 1.365 *μ*g·g^−1^, respectively. *β*-caryophyllene could not be detected in stems. The concentration of *p*-cymene, thymol, and neryl acetate in stems were within the range of 1.79 ± 0.034∼5.90 ± 0.659 *μ*g·g^−1^, 5.48 ± 0.043∼19.10 ± 1.729 *μ*g·g^−1^, and 12.51 ± 0.115∼40.94 ± 0.182 *μ*g·g^−1^, respectively. Only thymol could be detected in roots, and the contents ranged from 5.42 ± 0.547 to 32.60 ± 0.409 *μ*g·g^−1^. In a previous study, it has been found that the compounds from the volatile oils extracted from *Eupatorium fortunei* Turcz. with the three methods were different [21]. The treatment and processing of herbs also affect the composition and content of volatile oil from *Eupatorium fortunei* Turcz. [22].

All the constituents could be detected in leaves, and only thymol exists in roots. The order of the contents of four constituents in different parts was leaves > stems > roots. Volatile oil exists in glandular hairs, oil rooms, oil tubes, secretory cells, or resin ducts. Volatile oil exists in different parts of different plants. Some of them are found in the whole plant, while others are found in some organs of flowers, fruits, leaves, roots, or rhizomes. The location of volatile oil varies with plant species. Hence, one can see that the studied four compounds mainly existed in leaves of *Eupatorium fortunei* Turcz. Higher content of essential oil in leaf than other organs of a plant has been previously reported for other species [19, 23]. Based on that, it could be concluded that the leaves should be the best medicinal part of *Eupatorium fortunei* Turcz.

### 3.4. Dynamic Characteristics of Four Constituents in Different Growth Periods of Eupatorium fortunei Turcz

The concentrations change of *p*-cymene, thymol, neryl acetate, and *β*-caryophyllene in different parts of *Eupatorium fortunei* Turcz. with growth time were also analyzed. The contents of all constituents in leaf showed a trend of increase-decrease-increase-decrease with the plant growing. The content of *p*-cymene accumulated to its highest value on July 15. After an 80% reduction, the content rose again to 86% of the highest value approximately on September 15. Thymol reached its first peak value on August 15 and then reached the highest level on September 15. The highest value of thymol was about 6.5 times and 3.3 times higher than that on September 1 and October 1. The content of neryl acetate was the highest from July 1 to July 15, and it was also high on September 15, which is about 74% of the highest value. The yield of *β*-caryophyllene was the highest on July 1 and then decreased sharply. The content of *β*-caryophyllene increased again from 17.86 *μ*g·g^−1^ on September 1 to 44.38 *μ*g·g^−1^ on September 15. The contents of four constituents in the stem at each harvest stage showed different patterns. The yield of *p*-cymene was the highest on August 15. After a 63% reduction, *p*-cymene content rose again to a higher value on October 15. While the content of thymol was highest on April 15, it then decreased with the plant growth. Similarly, the content of neryl acetate reached the highest value on April 15 and had the second highest content on July 15, which was about 90% of the highest value. Only thymol could be detected in roots from June 1 to August 15, and the content was the highest on June 1.

In summary, the contents of four constituents in different parts of *Eupatorium fortunei* Turcz. reached the highest value in early July, followed by mid-September. These two time points could be considered as the best time to harvest the plant.

To the best of our knowledge, there are no studies regarding the relationship between the growth stages of *Eupatorium fortunei* Turcz. and the content of essential oil extracted from it. In other studies, it has been found that the contents of many biological active compounds in plants depend on growth time; the yields of compounds usually reach the highest at summer or flowering stages (July or August) [18, 19].

## 4. Conclusions

A quantitative determination method was developed and validated to assay the contents of *p*-cymene, thymol, neryl acetate, and *β*-caryophyllene in *Eupatorium fortunei* Turcz. by GC-MS/MS. The contents of the four constituents in samples from different parts were assayed and distribution was analyzed. All constituents could be detected in leaves. The contents order of constituents in different parts was leaves > stems > roots. From that, we could deduce that leaves should be the best medicinal part of *Eupatorium fortunei* Turcz. The growth stages of the plant strongly influence the content of four analytes in *Eupatorium fortunei* Turcz. The four constituents accumulated to the highest value in early July followed by mid-September. Accordingly, the best harvest time of *Eupatorium fortunei* Turcz. is early July and mid-September.

## Figures and Tables

**Table 1:** Target ion pair, reference ion pairs, and collision voltage of compounds.

Compounds	Target ions (m/z)	Collision voltage (eV)	Reference ions 1 (m/z)	Collision voltage (eV)	Reference ions 2 (m/z)	Collision voltage (eV)
*p*-Cymene	119 > 91.1	15	134 > 119.1	9	119 > 117.1	12
Thymol	135 > 91.1	18	150 > 135.1	9	135 > 115.1	15
Neryl acetate	93 > 77.1	12	93 > 91.1	12	93 > 51.1	24
*β*-Caryophyllene	93 > 77.1	12	93 > 91.1	9	91 > 65.1	15

**Table 2:** Validation of the optimized GC-MS/MS.

Compounds	Regression equation	*r*	LOD (ng·mL^−1^)	LOQ (ng·mL^−1^)	Precision (RSD%)	Recovery (%)	Repeatability (RSD%)	Stability (RSD%)
*p*-Cymene	*y* = 33388*x*−46.065	0.9995	6	12	3.38	105.31	1.00	1.11
Thymol	*y* = 19378*x*−523.59	0.9996	14	54	2.98	106.41	2.72	1.36
Neryl acetate	*y* = 8149*x*−441.05	0.9990	28	110	0.98	99.61	1.55	1.09
*β*-Caryophyllene	*y* = 8152*x*−327.55	0.9992	38	129	2.70	102.24	1.59	1.04

**Table 3:** Investigation of extraction conditions.

	*p*-Cymene	Thymol	Neryl acetate	*β-*Caryophyllene
Extraction method (*μ*g·g^−1^)				
Ultrasonic	3.67 ± 0.079	96.98 ± 0.759	47.49 ± 1.390	58.70 ± 0.642
Reflux	2.96 ± 0.137	77.59 ± 0.461	30.82 ± 0.227	10.49 ± 0.661

Extraction duration (*μ*g·g^−1^)				
15 min	2.07 ± 0.040c	61.60 ± 0.632d	42.22 ± 0.651d	28.53 ± 0.449d
30 min	3.67 ± 0.079b	96.98 ± 0.759c	47.49 ± 1.390c	58.70 ± 0.642c
45 min	4.01 ± 0.079a	107.06 ± 1.937b	60.16 ± 0.940b	65.36 ± 1.282b
60 min	4.12 ± 0.139a	111.80 ± 2.317a	67.32 ± 0.659a	70.64 ± 1.906a

Data are presented as mean ± standard deviation. Values with the same letter within each column are not significantly different (*p* < 0.05).

**Table 4:** The content of *p*-cymene, thymol, neryl acetate, and *β*-caryophyllene in different parts of *Eupatorium fortunei* Turcz.

Harvest date	*p*-Cymene (*μ*g·g^−1^)		Thymol (*μ*g·g^−1^)			Neryl acetate (*μ*g·g^−1^)		*β*-Caryophyllene (*μ*g·g^−1^)
	Leaf	Stem	Leaf	Stem	Root	Leaf	Stem	Leaf
April 15	—	1.80 ± 0.008c	39.19 ± 0.056g	19.10 ± 1.729a	—	—	40.94 ± 0.182a	—
May 1	1.51 ± 0.056g	1.85 ± 0.051c	46.51 ± 1.160g	13.00 ± 0.139b	—	—	—	13.36 ± 2.611e
May 15	—	2.70 ± 0.129bc	43.80 ± 0.406g	13.41 ± 0.410b	—	—	22.15 ± 6.623bc	—
June 1	7.22 ± 0.013e	3.77 ± 0.278bc	59.04 ± 1.227f	—	32.60 ± 0.409a	—	—	—
June 15	15.63 ± 0.325c	3.43 ± 0.310bc	86.68 ± 4.316c	—	5.56 ± 0.551c	48.58 ± 0.951c	—	105.55 ± 8.190b
July 1	10.73 ± 0.832d	5.20 ± 0.060ab	78.25 ± 1.503d	—	12.07 ± 0.554b	54.69 ± 2.065b	26.41 ± 0.266b	144.21 ± 1.365a
July 15	25.25 ± 2.600a	5.06 ± 0.170ab	68.32 ± 1.462e	—	5.64 ± 0.504c	59.90 ± 1.006a	37.42 ± 1.648a	96.55 ± 1.316b
August 1	20.93 ± 1.012b	5.25 ± 0.345ab	74.21 ± 3.726de	—	5.42 ± 0.547c	28.11 ± 0.149d	32.38 ± 4.046ab	67.45 ± 6.657c
August 15	8.11 ± 0.398e	5.90 ± 0.659a	113.77 ± 4.040b	—	5.49 ± 0.450c	—	—	20.31 ± 0.188e
September 1	5.06 ± 0.267f	4.18 ± 0.279ab	20.81 ± 0.688h	—	—	17.86 ± 0.452e	—	36.42 ± 1.665d
September 15	21.61 ± 0.006b	2.20 ± 0.173c	132.21 ± 3.169a	—	—	44.38 ± 1.773c	—	137.05 ± 2.760a
October 1	7.49 ± 0.308e	2.62 ± 0.279bc	40.76 ± 3.921g	—	—	13.77 ± 2.600ef	16.64 ± 4.196c	—
October 15	7.79 ± 0.180e	3.98 ± 0.166b	78.68 ± 0.573d	—	—	14.11 ± 1.807ef	12.51 ± 0.115c	—
November 1	3.48 ± 0.087f	1.79 ± 0.034c	63.24 ± 3.444ef	5.48 ± 0.043c	—	—	—	—

“—”, not detected. Data are presented as mean ± standard deviation. Values with the same letter within each column are not significantly different (*p* < 0.05).

## Data Availability

The data used to support the findings of this study are included within the article.
